# A systematic review and meta-analysis of imaging characteristics and upgrade rates in noninvasive lobular neoplasia of the breast

**DOI:** 10.1016/j.ejro.2025.100691

**Published:** 2025-10-15

**Authors:** Fatemeh Shakki Katouli, Negin Salehi, Faezeh Soveyzi, Mina Abedi, Parya Valizadeh, Hamed Ghorani, Seyedeh Melika Hashemi, Jayran Zebardast, Madjid Shakiba, Sadaf Alipour

**Affiliations:** a Department of Radiology, Arash Women Hospital,Tehran University of Medical Sciences,Tehran, Iran; b Student Research Committee, School of Pharmacy and Pharmaceutical Sciences, Isfahan University of Medical Sciences, Isfahan, Iran; c Department of Radiology, Mashhad University of Medical Sciences, Mashhad, Iran; d Systematic Review and Meta-analysis Expert Group (SRMEG), Universal Scientific Education and Research Network (USERN), Tehran, Iran; e Student's Research Committee, School of medicine, Mashhad University of Medical Sciences, Iran; f School of medicine, Tehran university of medical sciences, Tehran, Iran; g Department of Radiology, Shariati Hospital, Tehran University of Medical Sciences,Tehran, Iran; h Advanced Diagnostic and Interventional Radiology Research Center (ADIR), Tehran University of Medical Science, Tehran, Iran; i Department of Cognitive Linguistics, Institute for Cognitive Science Studies (ICSS), Tehran, Iran; j Breast Disease Research Center (BDRC), Tehran University of Medical Sciences, Tehran, Iran; k Department of Surgery, Arash Women’s Hospital, Tehran University of Medical Sciences, Tehran, Iran

**Keywords:** Non-invasive lobular neoplasia, Microcalcification, Breast cancer

## Abstract

**Background:**

Non-invasive lobular neoplasia (NLN) encompasses a range of lobular breast lesions that may precede invasive breast cancer. Microcalcifications detected through mammography play a crucial role in evaluating breast lesions and are often associated with NLN. This study focuses on the prevalence and significance of microcalcifications in NLN, noting that they can be the sole radiographic finding in many cases. While mammography is highly sensitive for detecting microcalcifications, it has limitations in diagnosing NLN, as some lesions may not show up on scans. Advanced imaging techniques like magnetic resonance imaging (MRI) offer improved diagnostic accuracy, particularly in dense breast tissue, but more research is needed for their routine use. Additionally, the risk of NLN progressing to malignant lesions highlights the importance of vigilant monitoring and management. This study aims to analyze the relationship between microcalcifications and NLN, addressing progression risks and implications for clinical practice.

**Method:**

This systematic review and meta-analysis (CRD42022346891) involved a comprehensive search of databases such as PubMed and Scopus from 2000 to 2023, using keywords related to lobular carcinoma and mammography. Eligible English-language studies included those reporting mammographic findings of pure NLN lesions confirmed by histopathologic evaluation and surgical excision. Exclusion criteria involved studies without surgical results or definitive imaging findings. Two independent reviewers assessed titles and abstracts, resolving discrepancies as needed. Data were systematically extracted using a standardized form, with the selection process depicted in a PRISMA flow diagram.

**Result:**

Meta-analysis of included studies revealed that the pooled proportion of any mammographic microcalcification among all NLN lesions was 0.70 (95 % CI: 0.63–0.76). Pure microcalcification (without an associated mass or distortion) was the most common presentation, with a pooled proportion of 0.67 (95 % CI: 0.60–0.74) among all lesions and 0.99 (95 % CI: 0.96–1.00) among lesions presenting with any microcalcification. The overall pooled upgrade rate to malignancy was 0.18 (95 % CI: 0.11–0.25), with a significantly higher rate for lobular carcinoma in situ (LCIS) at 0.22 (95 % CI: 0.15–0.30) compared to atypical lobular hyperplasia (ALH) at 0.06 (95 % CI: 0.01–0.14). Microcalcifications were present in the majority of upgraded lesions (pooled proportion: 0.78, 95 % CI: 0.69–0.87). A small but significant proportion of lesions (0.08, 95 % CI: 0.03–0.17) had no mammographic findings. All pooled estimates showed high heterogeneity. Sensitivity analysis confirmed the robustness of the results, while Egger's test indicated potential publication bias.

**Conclusion:**

In conclusion this study highlights the significant prevalence of microcalcifications in NLN cases and their critical role as a diagnostic feature. Despite their association with disease progression, microcalcifications are not reliable predictors of malignancy upgrade. Further evaluation is necessary to understand their clinical implications and improve management strategies for patients with NLN.

## 1 Introduction

According to the 5th edition Classification of Tumors of the Breast by the World Health Organization (WHO), the morphological spectrum of noninvasive lobular neoplasia (NLN) encompasses atypical lobular hyperplasia (ALH), classic lobular carcinoma in situ (classic LCIS), and two variants of LCIS, pleomorphic LCIS (P-LCIS) and florid LCIS (F-LCIS). Noninvasive lobular neoplasia refers to atypical epithelial proliferation comprising noncohesive cells due to loss or functional change of E-cadherin-mediated cell adhesion [1], [2]. The specific morphologic or molecular characteristics predictive of aggressive behavior in NLNs remain unknown [3], [4], [5].

The true incidence of LCIS is challenging (about 0.5–2.9 % of imaging-found lesions that were sampled by CNB or VAB). The increased utilization of advanced imaging techniques in breast cancer screening has likely contributed to the recently rising incidence of LCIS. Evidence suggests that NLN is associated with a higher risk of developing invasive breast cancer, 7–12 times higher than the general population, both as a risk factor (developing breast cancer in either breast) and a nonobligate precursor lesion. Furthermore, genomic studies support the notion that certain lobular neoplasia lesions may act as precursors with the potential to progress directly to invasive carcinoma [2], [6], [7].

There are two approaches in the interpretation of imaging modalities searching for NLN. Some authors believe in NLN as a mammographically occult lesion. In the presence of mammographic microcalcifications, they are found separately from the lobular neoplastic tissue as an incidental finding. Conversely, others believe in microcalcification as the common mammographic finding [8]. In rare cases of mammographically occult NLN, the lesions are found on ultrasound as a breast mass or in MRI as non-mass enhancement [7].

There is considerable debate surrounding the optimal management of lobular neoplasia. The recommended management varies from follow-up in cases of pure NLN with concordant radiology-pathology to surgery in selective cases. The upgrade rate of NLN to malignancy varies in the literature, with a higher rate for P-LCIS. Microinvasive carcinomas have been observed in association with ALH and classic LCIS, although this occurrence is not common. Findings of a systematic review and meta-analysis by Shehata et al. demonstrated relatively low pooled upgrade rates—particularly in radiologic-pathologic concordant lesions—supporting a more individualized and conservative management approach in select cases [9]. Together, these data contribute to a nuanced view of lobular neoplasia: while not obligate precursors of cancer, these lesions mark a significantly increased lifetime risk that demands careful multidisciplinary evaluation and tailored follow-up strategies.

In this study, we conducted a systematic review and meta-analysis of mammographic findings of pathologically-proven noninvasive lobular neoplasia (surgical pathology) and the upgrade rate regarding mammographic presentation.

## 2 Method

### 2.1 Search strategy and data collection

This study was registered in the International Prospective Register of Systematic Reviews (Registration Number: CRD42022346891). The databases, including PubMed, Scopus, ISI Web of Science, and Google Scholar, were searched from 2000 to 2023 for the relevant articles. The search strategy was all the possible combinations of ”lobular carcinoma”, “ breast carcinoma in situ”, ”non-invasive lobular carcinoma”, “breast neoplasm “, ‘mammography’, and ‘’diagnostic imaging’’. Included articles were reviewed and then screened for the selection of relevant articles.

### 2.2 Inclusion/exclusion criteria

All studies, including registries, descriptive studies, and analytic studies consisting of prospective or retrospective cohort studies, cross-sectional, case-control, and controlled trials published in English were considered. Review studies and articles irrelevant to the subject were excluded. Relevant articles were those that reported the mammographic findings of histologically confirmed pure NLN lesions following surgical excision. The studies were excluded if the upgraded cases were excluded or if the mammographic findings and surgical results could not be matched for each case.

### 2.3 Variables and measures

Mammographic features of NLN were classified as microcalcification, mass, mass with microcalcification, architectural distortion, architectural distortion with microcalcification, and asymmetry. The upgrade rate was extracted and reported into three categories, including the upgrade rate in LCIS, ALH, and NL lesions. Moreover, mammographic findings of upgraded lesions were extracted. Two meta-analyses were performed, one considered the studies that reported mammographic findings in NLN lesions, and the other included studies that reported the upgrade rate and described the mammographic findings in the upgraded cases.

### 2.4 Data extraction and quality assessment

Upon completion of the search process, duplicates were identified, manually re-checked="checked" value="1", and removed. Citations identified from literature searches and reference list checking were imported to EndNote. Two reviewers independently examined titles and abstracts for selected studies that fulfilled the inclusion criteria. Subsequently, the same two reviewers evaluated the full texts. After that, all potentially eligible records were retrieved, and the final remaining articles were included in the review. Any disagreements regarding data extraction were resolved through discussion and, when necessary, consultation with a third review author. Two reviewers independently collected data from each study and completed the data extraction form. Risk of bias was evaluated using the Quality Assessment of Diagnostic Accuracy Studies 2 (QUADAS-2) tool using four domains: patient selection, index test, reference standard, and flow/timing. Both reviewers independently completed the assessment, resolving discrepancies by consensus.

### 2.5 Ethics and dissemination

The data was extracted from published articles, and no human or animal rights were compromised during this process.

## 3 Result

### 3.1 Study selection

**Fig. 1:**
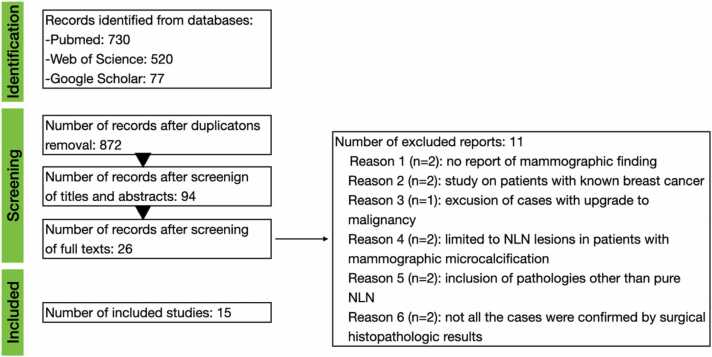
PRISMA flowchart.

The search process was completed on 14/08/2022 and updated on 17/1/2023. Two authors independently screened all articles. The initial number of papers identified was 1933. After removing duplicates, 872 articles were screened by titles and abstracts. In the second round, 94 articles underwent full-text screening. The application of eligibility criteria to 26 studies included 15 studies published online between 2001 and 2021. Fig. 1 illustrates the study selection process.

### 3.2 Study characteristics

Table 1, Table 2 presents the characteristics extracted from the 15 included studies, all employing a retrospective design. One study from Italy was multicenteric, while the others were single-center. The included studies originated from 15 research groups across six countries, reporting 1203 patients who underwent surgical excision of NLN with available mammographic findings. One study (by Ibrahim et al.) was excluded from the meta-analysis of mammographic findings due to the inability to match the mammographic findings with CNB results. Additionally, two studies were excluded from the meta-analysis of mammogram findings in upgraded cases (by Capobianco et al. and Scoggins et al.) due to the inability to describe the mammographic findings in upgraded cases.

**Table 1:** Quality assessment of included articles.

Authors	1	2	3	4	5	6	7	8
S. Bianchi 2014	Yes	Yes	Yes	Yes	N/A	N/A	Yes	Yes
J. Cangiarella 2007	Yes	Yes	Yes	Yes	N/A	N/A	Yes	N/A
G. Capobianco 2014	Yes	Yes	Yes	Yes	N/A	N/A	Yes	N/A
S.V. Destounis 2011	Yes	Yes	Yes	Yes	Yes	No	Yes	N/A
K.M. Flegg 2010	Yes	Yes	Yes	Yes	N/A	N/A	Yes	N/A
G.M. Crisi 2003	Yes	Yes	Yes	Yes	N/A	N/A	Yes	N/A
G.Y. Yoon 2021	Yes	Yes	Yes	Yes	Yes	Yes	Yes	Yes
H. Hwang 2008	Yes	Yes	Yes	Yes	Yes	Yes	Yes	N/A
N. Ibrahim 2011	Yes	Yes	Yes	Yes	N/A	N/A	Yes	Yes
L.Q. Chang Sen 2016	Yes	Yes	Yes	Yes	Yes	No	Yes	Yes
V. Londero 2008	Yes	Yes	Yes	Yes	N/A	N/A	Yes	Yes
B. Niell 2012	Yes	Yes	Yes	Yes	Yes	No	Yes	Yes
D. O'Driscoll 2001	Yes	Yes	N/A	Yes	N/A	N/A	Yes	N/A
M. Scoggins 2013	Yes	Yes	Yes	Yes	N/A	N/A	Yes	N/A
Barry Amos 2016	Yes	Yes	Yes	Yes	N/A	N/A	Yes	N/A

1. Were the criteria for inclusion in the sample clearly defined? 2. Were the study subjects and the setting described in detail? 3. Was the exposure measurement valid and reliable? 4. Were objective, standard criteria used for measurement of the condition? 5. Were confounding factors identified? 6. Were strategies to deal with confounding factors stated? 7. Were the outcomes measured validly and reliably? 8. Was an appropriate statistical analysis used?

**Table 2:** Characteristics of included articles.

First Author Year of Publication	Country	Type of study	Single center vs Multicentric	Total No. of Patients	Reference Standard	Inclusion in meta-analysis of mammographic findings	Inclusion in meta-analysis of mammogram findings in upgraded cases
D. O'Driscoll 2001	UK	Retro	Single center	7	Surgical pathology	Yes	Yes
G.M. Crisi 2003	USA	Retro	Single center	16	Surgical pathology	Yes	Yes
H. Hwang 2008	USA	Retro	Single center	87	Surgical pathology	Yes	Yes
V. Londero 2008	Italy	Retro	Single center	35	Surgical pathology	Yes	Yes
K.M. Flegg 2010	Australia	Retro	Single center	9	Surgical pathology	Yes	Yes
S.V. Destounis 2011	USA	Retro	Single center	64	Surgical pathology	Yes	Yes
N. Ibrahim 2011	Canada	Retro	Single center	89	Surgical pathology	No*	Yes
B. Niell 2012	USA	Retro	Single center	51	Surgical pathology	Yes	Yes
S. Bianchi 2014	Italy	Retro	Multicentric	149	Surgical pathology	Yes	Yes
G. Capobianco 2014	Italy	Retro	Single center	65	Surgical pathology	Yes	No +
M. Scoggins 2013	USA	Retro	Single center	31	Surgical pathology	Yes	No +
G.Y. Yoon 2021	Korea	Retro	Single center	55	Surgical pathology	Yes	Yes
Barry Amos 2016	USA	Retro	Single center	63	Surgical pathology	Yes	Yes
J. Cangiarella 2007	USA	Retro	Single center	38	Surgical pathology	Yes	Yes
L.Q. Chang Sen 2016	USA	Retro	Single center	444	Surgical pathology	Yes	Yes

* Inability to match the mammographic findings with histopathologic results for each lesion + Inability to describe the mammographic findings in the subgroup of upgraded cases.

### 3.3 Descriptive analysis

Table 3 summarizes the data on mammographic findings of NLN lesions. Among 1108 cases with NLN, 772 cases (69.67 %) exhibited mammographic microcalcifications. Microcalcification was the sole finding in 718 cases (64.80 %). Negative mammograms were reported in 87 cases (7.85 %).

**Table 3:** Data on mammographic findings of NLN lesions.

First Author Year of Publication	Total number of cases	Total number of cases with MC	Only MC	Mass	Mass/MC	Distortion	Distortion/MC	Asymmetry	Negative
D. O'Driscoll 2001	7	6	4	1	2	0	0	0	0
G.M. Crisi 2003	16	11	11	5	0	0	0	0	0
H. Hwang 2008	87	60	59	27	0	0	1	0	0
V. Londero 2008	35	22	19	3	3	0	0	0	10
K.M. Flegg 2010	9	7	7	0	0	1	0	1	0
S.V. Destounis 2011	64	43	39	9	2	2	2	0	10
B. Niell 2012	51	33		8	0	0	0	0	10
S. Bianchi 2014	149	124	124	25	0	0	0	0	0
G. Capobianco 2014	62	47	44	11	2	0	1	0	4
M. Scoggins 2013	31	25	24	0	0	2	1	1	3
G.Y. Yoon 2021	52	19	19	13	0	0	0	0	20
Barry Amos 2016	63	37	37	8	0	0	0	0	18
J. Cangiarella 2007	38	31	31	1	0	0	0	1	5
L.Q. Chang Sen 2016	444	307	300	119	7	5	0	6	7

Table 4 summarizes the data on NLN lesions with upgrade to malignancy after surgical excision. Among 1167 included cases (557 LCIS, 553 ALH, and 59 LN), 165 lesions (14.13 %) demonstrated upgrade to malignancy (115 LCIS: 20.64 %, 40 ALH: 7.23 %, and 10 LN: 16.94 %). In upgraded cases, Microcalcification was the most frequently reported mammographic finding (117 lesions: 70.09 %). Furthermore, microcalcification was the only mammographic finding in 105 lesions (63.63 %). Radiologic-pathologic discordance in upgraded cases was reported in four studies (Hwang et al., Neil et al., Amos et al., Chang et al.) for a total of 9 LCIS and 4 ALH lesions.

**Table 4:** Data on NLN lesions with upgrade to malignancy after surgical excision.

First Author Year of Publication	Total number	Upgrade rate	Upgrade rate of NLN subtypes			Mammographic finding in upgrade cases		
			LCIS	ALH	LN	Total number of cases with MC	Only MC	Without MC
D. O'Driscoll 2001	7	3/7	3/7	0	0	3	2	0
G.M. Crisi 2003	16	2/16	1/8	0/3	1/5	1	0	1
H. Hwang 2008	87	10/87	9/39	1/48	0	9	8	1
V. Londero 2008	28	13/28	12/20	1/8	0	7	5	6
K.M. Flegg 2010	9	2/9	2/5	0/4	0	2	2	0
S.V. Destounis 2011	64	21/64	21/64	0	0	16	14	5
N. Ibrahim 2011	89	30/89	17/43	11/40	2/6	24	24	6
B. Niell 2012	51	8/51	7/35	1/16	0	5	2	2
S. Bianchi 2014	149	25/149	12/59	13/90	0	23	23	2
G. Capobianco 2014	65	7/65	7/65	0	0	0	0	0
M. Scoggins 2013	22	3/22	3/22	0	0	0	0	0
G.Y. Yoon 2021	55	9/55	9/55	0	0	7	6	2
Barry Amos 2016	43	7/43	0	0	7/43	5	5	2
J. Cangiarella 2007	38	3/38	2/20	1/18	0	1	1	2
L.Q. Chang Sen 2016	444	22/444	10/106	12/338	0	14	13	8

### 3.4 Meta-analysis

We implemented meta-analyses for the estimation of pooled proportions based on the included studies. These proportions included total microcalcification, pure microcalcification, microcalcification associated with mass, microcalcification associated with distortion, mass, distortion, and asymmetry ratios. In addition, the proportion of lesions with negative mammographic findings was calculated. In addition, among all patients with microcalcification, the pooled estimate of pure microcalcification, microcalcification associated with mass, and microcalcification associated with distortion was calculated. Similar proportions were calculated among patients with ALH and LCIS. In addition, similar proportions were calculated among patients who showed an upgrade of the lesion. In addition, the proportion of upgrades was calculated among all patients.

Pooled estimate of all forms of microcalcification was 0.70 [95 % Confidence interval (CI)= 0.63–0.76, I^2^= 74 %, P < 0.001]. Pooled estimate of pure microcalcification among all patients was 0.67 [95 % CI= 0.60–0.74, I^2^= 76.9 %, P < 0.001]. Pooled estimate of mass among all patients was 0.15 [95 % CI= 0.09–0.20, I^2^= 60.07 %, P < 0.001]. Totally, 0.08 of patients did not show any findings in their mammogram [95 % CI= 0.03–0.17, I^2^= 92.02 %, P < 0.001]. In addition, the pooled estimate of pure microcalcification among all patients with any form of microcalcification was 0.99 [95 % CI= 0.96–1, I^2^= 56.07 %, P = 0.01]. Pooled estimate of upgrade rate was 0.18 [95 % CI= 0.11–0.25, I^2^= 99.93 %, P < 0.001]. Pooled estimate of LCIS, ALH, and LN among all patients were 0.68, 0.21, and 0.03 [95 % CI= 0.46–0.87, 0.06–0.41, and 0–0.13; respectively, I^2^= 97.94 %, 0.97.92 % and 0.96.29 %; respectively, all Ps< 0.001]. Pooled estimate of upgrade among LCIS, ALH, and LN were 0.22, 0.06, and 0.16 [95 % CI= 0.15–0.30, 0.01–0.14, and 0.06–0.29; respectively, I^2^= 72 %, 0.72.86 % and 0 %; respectively, P < 0.001, < 0.001, and 0.57; respectively]. Pooled estimate of all forms of microcalcification among upgraded lesions was.078 [95 % CI= 0.69–0.87, I^2^= 20.81 %, P = 0.23]. Pooled estimate of mammographic findings among LCIS and ALH lesions has been mentioned in Table 5.

**Table 5:** Pooled estimate of proportions related to imaging findings and upgrade rate among all lesions and in LCIS, ALH, and LN lesion subgroups.

Group	Proportion	Pooled Estimation	95 % CI	I^2^ (%)	P-Value [I^2^]	Tau^2^
All Lesions	Any Form of Microcalcification	0.70	0.63–0.76	73.96	<0.001	0.04
	Pure Microcalcification	0.67	0.60-.074	76.93	<0.001	0.05
	Microcalcification Associated with Mass	0	0–0.02	46.70	0.03	0.01
	Microcalcification Associated with Distortion	0	0	19	0.24	0
	Mass	0.15	0.09–0.20	78.36	<0.001	0.05
	Distortion	0	0	9.14	0.35	0
	Asymmetry	0	0	0	0.56	0
	No Finding (Negative Imaging)	0.08	0.03–0.17	92.02	<0.001	0.17
	Upgrade	0.18	0.11–0.25	85.99	<0.001	0.09
	LCIS	0.68	0.46–0.87	97.94	<0.001	0.70
	ALH	0.21	0.06–0.41	97.92	<0.001	0.69
	LN	0.03	0–0.13	96.29	<0.001	0.38
Lesions with Microcalcification	Pure Microcalcification	0.99	0.96–1	56.07	0.01	0.03
	Mass Associated with Microcalcification	0.01	0–0.02	48.14	0.03	0.02
	Distortion Associated with Microcalcification	0	0	19.50	0.25	0
LCIS Lesions	Upgrade	0.22	0.15–0.30	72.00	<0.001	0.07
	Any Form of Microcalcification	0.66	0.58–0.75	60.68	<0.001	0.05
	Pure Microcalcification	0.64	0.54–0.73	59.21	0.01	0.05
	Microcalcification Associated with Mass	0	0–0.02	13.45	0.32	0.01
	Microcalcification Associated with Distortion	0	0–0.01	0	0.94	0
	Mass	0.12	0.04–0.23	82.29	<0.001	0.14
	Distortion	0	0–0.01	0	0.81	0
	Asymmetry	0	0	0	0.61	0
	No Finding	0.1	0.03–0.20	82.90	<0.001	0.15
LCIS Lesions with Microcalcification	Pure Microcalcification	0.98	0.94–1	6.18	0.38	0
	Microcalcification Associated with Mass	0	0–0.03	0	0.44	0
	Microcalcification Associated with Distortion	0	0–0.03	0	0.99	0
ALH Lesions	Upgrade	0.06	0.01–0.14	72.86	<0.001	0.06
	Any Form of Microcalcification	0.78	0.69–0.86	18.23	0.29	0.01
	Pure Microcalcification	0.79	0.54–0.97	63.66	0.02	0.15
	Microcalcification Associated with Mass	0	0–0.06	42.91	0.12	0.06
	Microcalcification Associated with Distortion	0	0	0	0.93	0
	Mass	0.16	0.10–0.24	13.89	0.32	0.01
	Distortion	0	0	0	0.54	0
	Asymmetry	0	0	0	0.96	0
	No Finding	0	0–0.05	57.47	0.03	0.05
ALH Lesions with Microcalcification	Pure Microcalcification	1	0.88–1	51.80	0.07	0.12
	Microcalcification Associated with Mass	0	0–0.12	51.80	0.07	0.12
	Microcalcification Associated with Distortion	0	0	0	0.93	0
LN Lesions	Upgrade	0.16	0.06–0.29	0	0.57	0
Upgraded Lesions	Any Form of Microcalcification	0.78	0.69–0.87	20.81	0.23	0.02
	Pure Microcalcification	0.64	0.52–0.74	0	0.67	0
	Microcalcification Associated with Other Findings	0.04	0–0.11	0	0.88	0
	No Finding	0.22	0.13–0.31	20.81	0.23	0.02

The most important proportions of the study include total microcalcifications among all lesions, pure microcalcification among all lesions, pure microcalcification among all lesions with microcalcification, upgrade rate among all lesions, lesions without any imaging finding (negative finding lesions) among upgraded lesions, total microcalcification among upgraded lesions, pure microcalcification of upgraded lesions, total microcalcifications among LCIS lesions, pure microcalcification in LCIS lesions, upgrade rate among LCIS lesions, total microcalcifications among ALH lesions and upgrade rate among ALH lesions. We performed subgroup analysis, meta-regression, sensitivity analysis, and publication bias assessments for these proportions (Table 6). As we can see, most of the pooled proportions show high heterogeneity with statistically significant P-values. So, we consider subgroup analysis for finding probable sources of heterogeneity. Among all possible reasons that could explain the heterogeneity (including imaging method, geographical region, study sample size, etc), we could perform subgroup analysis based on study sample size [studies with sample size ≤50 vs. >50]. In addition, we performed meta-regression considering study sample size as an independent variable and important ratios as a dependent variable. In all meta-regression analyses, the study sample size did not show a statistically significant P-value in meta-regressions (All P-Values>0.1) (Table 6). Classification of studies based on sample size (≤50 vs. >50) showed a relatively different pattern. In some ratios, heterogeneity was not seen in pooled estimates of ratios extracted from studies with sample sizes equal to or lower than 50, while the pooled estimate of ratios extracted from studies with sample sizes more than 50 showed statistically significant heterogeneity (Table 6).

**Table 6:** Meta-regression, subgroup analysis, sensitivity analysis, and publication bias results for important ratios of the study.

	Meta-Regression [Considering Study Sample Size as Independent Variable]		Subgroup Analysis Based on Study Sample Size				Sensitivity Analysis		Publication Bias	
			≤ 50		> 50		Min-Max of Pooled Ratios During Leave-One-Out Procedure	Main Pooled Estimate of The Ratio [95 % CI]	Egger Test	
	Coefficient	P-Value	Ratio [95 % CI]	I^2^ [P-Value]	Ratio [95 % CI]	I^2^ (%) [P-Value]			Bias	P
Total Microcalcifications among All Lesions	<0.001	0.95	0.76 [0.68–0.83]	0 [0.5]	0.67 [0.58–0.75]	84.1 [<0.001]	0.68–0.72	0.7 [0.63–0.76]	0.66	< 0.001
Pure Microcalcification among All Lesions	<0.001	0.95	0.71 [0.60–0.81]	35.4 [0.17]	0.65 [0.55–0.74]	76.9 [<0.001]	0.65–0.70	0.67 [0.60–0.74]	0.66	< 0.001
Pure microcalcifications among all lesions containing microcalcifications	<0.001	0.75	0.97 [0.88–1]	49.1 [0.08]	0.98 [0.96–1]	56.1 [0.01]	0.95–0.97	0.99 [0.96–1]	0.99	< 0.001
Upgrade Rate among All Lesions	<-0.001	0.12	0.20 [0.10–0.32]	63.5 [0.01]	0.17 [0.09–0.26]	90.9 [<0.01]	0.16–0.19	0.18 [0.11–0.25]	0.15	0.003
Lesions without Any Imaging Finding [Negative Finding Lesions] among Upgraded Lesions	<-0.005	0.29	0.33 [0.13–0.55]	1.9 [0.40]	0.21 [0.12–0.30]	19.3 [0.28]	0.19–0.25	0.22 [0.13–0.31]	0.26	0.014
Total Microcalcification among Upgraded Lesions	0.004	0.29	0.67 [0.45–0.87]	1.9 [0.4]	0.79 [0.70–0.88]	19.3 [0.28]	0.75–0.81	0.78 [0.69–0.87]	0.74	< 0.001
Pure Microcalcification of Upgraded Lesions	<-0.002	0.72	0.56 [0.32–0.78]	7.8 [0.36]	0.66 [0.54–0.77]	0 [0.87]	0.61–0.68	0.64 [0.52–0.74]	0.57	< 0.001
Total Microcalcifications among LCIS Lesions	<-0.008	0.55	0.70 [0.62–0.78]	0 [0.48]	0.62 [0.46–0.76]	85 [<0.001]	0.65–0.70	0.66 [0.58–0.75]	0.64	< 0.001
Pure Microcalcification in LCIS Lesions	<-0.001	0.46	0.68 [0.59–0.77]	0 [0.67]	0.57 [0.37–0.76]	59.2 [0.01]	0.62–0.68	0.64 [0.54–0.73]	0.61	< 0.001
Upgrade Rate among LCIS Lesions	<-0.001	0.20	0.27 [0.16–0.38]	60.7 [0.01]	0.17 [0.10–0.26]	75.3 [<0.001]	0.19–0.23	0.22 [0.15–0.30]	0.20	0.004
Total Microcalcifications among ALH Lesions	<-0.001	0.48	0.83 [0.59–0.99]	35 [0.19]	0.71 [0.67–0.76]	18.2 [0.29]	0.76–0.82	0.78 [0.69–0.86]	0.73	< 0.001
Upgrade Rate among ALH Lesions	<-0.002	0.23	0.04 [0–0.13]	0 [0.97]	0.10 [0.02–0.21]	89.5 [<0.001]	0.03–0.08	0.06 [0.01–0.14]	0.11	0.018

The leave-one-out sensitivity analysis demonstrated the robustness of the pooled effect size estimates for proportions. When each of the studies was excluded individually, all recalculated pooled estimates remained within the 95 % CI of the original proportion pooled estimate, indicating that no single study disproportionately influenced the overall results (Table 6).

For the evaluation of publication bias, Egger’s regression test was performed for the mentioned ratios that indicated significant small-study effects (P-values < 0.001), suggesting potential publication bias (Table 6). Visual inspection of the funnel plot suggested asymmetry, with smaller studies tending to report larger effect sizes. This observation was supported by Egger’s regression test, which indicated significant small-study effects.

**Fig. 2:**
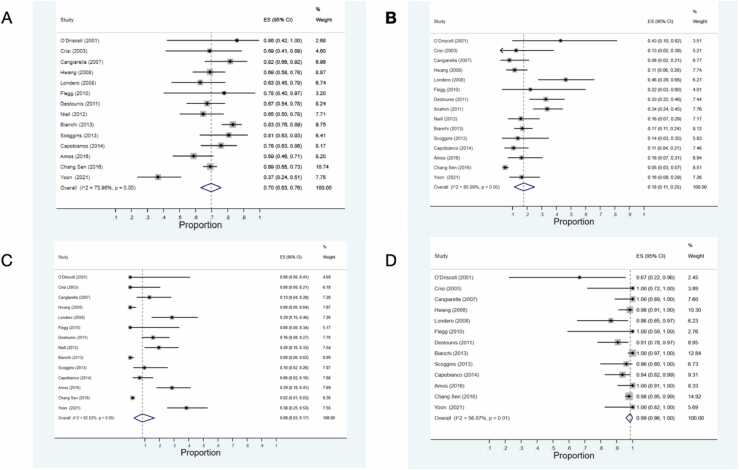
Forest Plots; A: Proportion of any forms of microcalcification among all lesions, B: Upgrade proportion among all lesions, C: Proportion of negative imaging finding among all lesions, D: Proportion of pure microcalcification among all forms of microcalcifications.

**Fig. 3:**
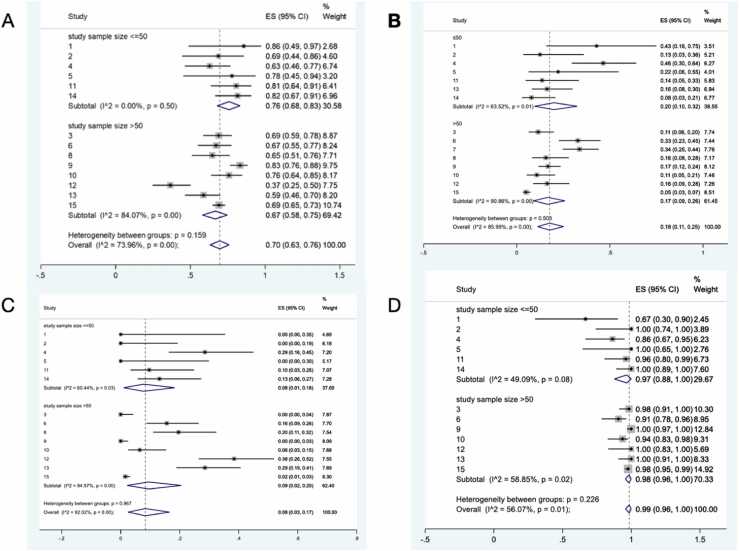
Subgroup analysis between two groups of studies with < =50 patients versus > 50 patients; A: Proportion of any forms of microcalcification among all lesions, B: Upgrade proportion among all Lesions, C: Proportion of negative imaging finding among all lesions, D: Proportion of pure microcalcification among all forms of microcalcifications.

**Fig. 4:**
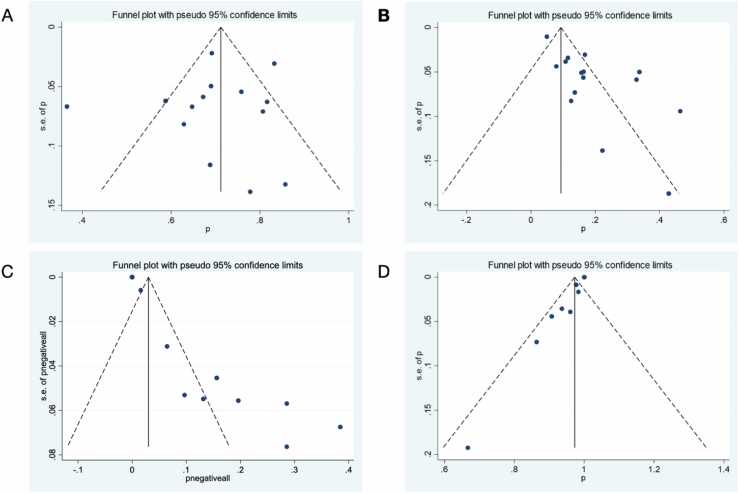
Funnel Plots; A: Proportion of any forms of microcalcification among all lesions, B: Upgrade proportion among all lesions, C: Proportion of negative imaging finding among all lesions, D: Proportion of pure microcalcification among all forms of microcalcifications.

Fig. 2, Fig. 3, Fig. 4 demonstrates forest plots, subgroup analysis, and funnel plots for the selected proportions, including any forms of microcalcification among all lesions, upgrade proportion among all lesions, proportion of negative imaging findings among All Lesions, and proportion of pure microcalcification among all forms of microcalcifications.

## 4 Discussion

This study observed that microcalcifications were present in 69.67 % of NLN cases. Notably, microcalcifications were the sole radiographic finding in over 50 % of NLN cases (specifically, 64.80 %). The pooled analysis revealed a statistically significant association between NLN lesions and mammographic microcalcifications, with a z-value of 25.88 and a p-value of 0.00. These findings prove that microcalcifications are a common mammographic feature in NLN lesions, reinforcing their diagnostic relevance in assessing breast pathologies. Furthermore, microcalcifications emerged as the predominant mammographic finding in NLN lesions that subsequently progressed to invasive lesions in surgical specimens, observed in 70.09 % of these cases.

However, it is essential to differentiate between types of microcalcifications and describe the anatomical distribution, as their morphology and distribution influence upgrade risk. For instance, in a study on 246 patients with suspicious microcalcifications on mammogram, PPVs of morphology descriptors were as follows: amorphous 7.9 %, coarse heterogeneous 17.8 %, fine pleomorphic 63.2 %, fine linear/fine linear branching 100 % (p < 0.001). PPVs of distribution descriptors were as follows: regional 8.8 %, grouped 14.3 %, linear 87.5 %, segmental 63.6 % (p < 0.001). For morphology and distribution descriptors combinations, PPVs for amorphous/regional and amorphous/grouped microcalcifications were 4.2 and 7.6 %, respectively. The PPV for fine pleomorphic/linear or segmental was 93.8 % [10]. Recent evidence also suggests that contrast-enhanced mammography may improve the characterization of suspicious calcifications, adding functional information to morphology-based assessment and thereby refining malignancy risk stratification [11]. Our analysis did not stratify microcalcifications by subtype, which limits the ability to refine or upgrade risk prediction based on calcification morphology. Future studies should aim to address this gap.

This aligns with the comprehensive meta-analysis by Shehata et al. (2020), which evaluated 65 studies to assess the risk of upgrade to malignancy following a core needle biopsy (CNB) diagnosis of LN [9]. In their analysis of 65 studies, Shehata et al. found that the risk of upgrade to any malignancy (invasive carcinoma or ductal carcinoma in situ) varied widely, ranging from 0 % to 45 %. However, focusing on 16 studies that met stringent quality criteria—excluding variant LCIS, imaging-discordant lesions, and ensuring outcome reporting for at least 70 % of lesions—they reported pooled upgrade risks as follows:

• All LN: 3.1 % (95 % CI, 1.8 %-5.2 %)

• ALH: 2.5 % (95 % CI, 1.6 %-3.9 %)

• LCIS: 5.8 % (95 % CI, 2.9 %-11.3 %)

For upgrades specifically to invasive malignancy, the pooled risks were:

• All LN: 1.3 % (95 % CI, 0.7 %-2.4 %)

• ALH: 0.4 % (95 % CI, 0.0 %-4.2 %)

• LCIS: 3.5 % (95 % CI, 2.0 %-5.9 %)

Their findings indicated a low risk of upgrade to malignancy for LN, particularly for ALH, suggesting that imaging surveillance could be a viable alternative to surgical management in selected cases [9]. In comparison, our observed rate of progression for NLN fell within this expected range, further supporting that while microcalcifications are common, the absolute risk of upgrade remains relatively low. This reinforces the importance of integrating imaging findings with histopathology and clinical risk factors, rather than relying solely on the presence of calcification.

Additionally, the detection of LN in MRI-guided core biopsies has been associated with higher upgrade rates, suggesting that the modality of detection may also play a role in management decisions [12].

Despite the prevalence of microcalcifications among NLN cases and more advanced disease states, it is essential to note that the presence of microcalcifications does not serve as a reliable predictor for the upgrade to malignancy. This distinction is crucial, as frequency does not equate to predictive value. While microcalcifications are the dominant mammographic feature, their role is primarily diagnostic rather than prognostic. This underscores the necessity for a comprehensive assessment that includes radiologic-pathologic correlation and consideration of patient-specific risk factors.

Moreover, the existing literature highlights the critical importance of microcalcification detection as a key biomarker for the early diagnosis of breast cancer. This finding underscores the potential of microcalcifications to serve as an important diagnostic tool and an indicator of malignancy in the context of breast imaging [13]. Certain neoplasms may present exclusively as microcalcifications, emphasizing the role of these findings in malignancy detection. Notably, mammography demonstrates continued efficacy in identifying such malignancies, even in patients with dense breast tissue, where the sensitivity of imaging techniques is often compromised. This underscores the importance of microcalcification assessment in the comprehensive evaluation of breast lesions [14]. Studies have shown that the sensitivity of mammography in detecting microcalcification-associated malignancies remains high, even in women with dense breasts. A meta-analysis by Kolb et al. found that the sensitivity of mammography for detecting microcalcifications was 87 %, regardless of breast density [15]. Similarly, a retrospective study by Stomper et al. reported that mammography had a sensitivity of 92 % for detecting DCIS, with no significant difference between women with dense and non-dense breasts [16]. Analyzing NLN lesions with negative mammograms revealed a pooled effect size of 0.08, accompanied by a 95 % confidence interval ranging from 0.03 to 0.17. This finding indicates that a small yet statistically significant proportion of NLN lesions can present with negative mammographic results. This underscores the limitations of mammography as a solitary screening tool for NLN detection.

The systematic review and meta-analysis conducted by Michael Faheem and colleagues underscored the significance of magnetic resonance imaging (MRI) as an adjunctive imaging modality in individuals with dense breast tissue who exhibit negative mammography results [17]. The study highlights the potential of MRI to enhance diagnostic accuracy and facilitate early detection of breast pathology in this patient population. In this study, the diagnostic rate of breast cancer was observed to be 16.6 cases per 1000 routine MRI examinations and 6.8 cases per 1000 random MRI examinations. These findings suggest a high-risk setting for routine MRI evaluations and a medium-risk context for random MRIs, highlighting the varying prevalence of breast cancer detection based on the examination strategy employed [17]. It offers enhanced lesion characterization through multiparametric assessment, which includes T1-weighted contrast-enhanced imaging, T2-weighted, ultrafast, and diffusion-weighted imaging. This comprehensive approach allows for better discrimination between benign and malignant breast lesions, particularly in patients with inconclusive mammographic findings [18]. In an investigation conducted by Rachel F. Brem et al. involving 26 individuals diagnosed with invasive lobular carcinoma, MRI was identified as the most sensitive diagnostic modality for this specific type of cancer, demonstrating a sensitivity rate of 83 % when compared to ultrasound and mammography. Notably, MRI could identify lesions that were not detectable by mammography, primarily due to the application of contrast enhancement, thereby underscoring the importance of MRI in the comprehensive evaluation of invasive lobular carcinoma [19].

According to the analyses conducted in our study, the rate of progression of NLN to malignant lesions demonstrated a statistically significant difference from zero. This finding underscores the potential for NLN to progress to carcinomatous lesions, highlighting the clinical implications associated with the monitoring and management of these lesions. Our observed progression rate was comparable to the long-term data reported by Jannah Baker et al. and others, indicating that while absolute progression is relatively low, the long-term risk remains clinically meaningful.

In the study by Jannah Baker et al., the 10-year cumulative incidence of LCIS was 11.3 %, with a 20-year cumulative incidence of 19.7 %. Additionally, the 10-year incidence of lobular neoplasia was reported to be 17 %. These findings contribute to understanding the long-term risk associated with these conditions, similar to our study [20]. Long-term studies have demonstrated that LCIS is associated with an increased risk of subsequent breast cancer. For instance, women with LCIS have about a 7–12 times higher risk of developing invasive cancer in either breast, necessitating regular breast cancer screening tests and follow-up visits with healthcare providers [21]. In a retrospective cohort study by Emily Vicks and colleagues, a notable increase in progression towards increased invasiveness was observed following CNB in a subset of suspicious cases. Specifically, higher rates of progression were identified in cases of LN and atypical ductal hyperplasia (ADH) compared to other pathological conditions [22]. Currently, there is a paucity of data available to accurately predict the progression of ALH and LCIS towards invasive disease based solely on mammographic findings. Therefore, further investigations are essential to improve understanding and refine predictive models [23]. From a clinical standpoint, our findings support a nuanced management approach: (1) surgical excision may be warranted when LN is associated with imaging-pathology discordance, pleomorphic or suspicious microcalcifications, or detection via MRI-guided biopsy; (2) imaging surveillance can be considered for concordant, low-risk findings such as amorphous calcifications or isolated ALH; and (3) microcalcifications alone should not trigger automatic excision but should prompt careful radiologic-pathologic correlation and individualized risk assessment.

## 7 Conclusion

In conclusion, this study demonstrates that microcalcifications are a prevalent and significant mammographic finding in non-invasive lobular neoplasia (NLN), potentially serving as a diagnostic tool for assessing breast pathologies. Although microcalcifications are common, they do not reliably predict the progression to malignancy. Continued research and the incorporation of advanced imaging techniques like MRI are essential to enhance diagnostic accuracy and patient management for individuals with NLN.

## CRediT authorship contribution statement

**Madjid Shakiba:** Methodology, Investigation, Formal analysis, Conceptualization. **Sadaf Alipour:** Formal analysis, Data curation, Conceptualization. **Fatemeh Shakki Katouli:** Visualization, Validation, Supervision, Software, Resources, Project administration, Methodology, Formal analysis, Data curation, Conceptualization. **Negin Salehi:** Validation, Supervision, Software, Investigation, Formal analysis, Conceptualization. **Faezeh Soveyzi:** Writing – review & editing, Writing – original draft, Visualization, Resources, Conceptualization. **Mina Abedi:** Methodology, Investigation, Formal analysis, Data curation. **Parya Valizadeh:** Resources, Project administration, Investigation, Formal analysis. **Hamed Ghorani:** Validation, Supervision, Software, Resources, Project administration, Conceptualization. **Seyedeh Melika Hashemi:** Resources, Project administration, Investigation, Formal analysis. **Jayran Zebardast:** Project administration, Methodology, Formal analysis.

## Ethical approval

This systematic review and meta-analysis does not involve human participants, animal subjects, or any form of direct experimentation requiring ethical approval. Therefore, approval from an ethics committee was not required. All studies included in this review were conducted in accordance with the ethical standards outlined in their respective publications.

## Funding

This study was not supported by any funding.

## Declaration of Competing Interest

The authors declare that there is no conflict of interests.
